# Novel 2-phenoxypyrido[3,2-*b*]pyrazin-3(4*H*)-one derivatives as potent and selective aldose reductase inhibitors with antioxidant activity

**DOI:** 10.1080/14756366.2019.1643336

**Published:** 2019-07-26

**Authors:** Xin Hao, Gang Qi, Hongxing Ma, Changjin Zhu, Zhongfei Han

**Affiliations:** a The State Key Laboratory of Medicinal Chemical Biology, Nankai University, Tianjin, PR China;; b Department of Applied Chemistry, Beijing Institute of Technology, Beijing, PR China;; c Faculty of Chemistry and Chemical Engineering, Yancheng Institute of Technology, Yancheng, PR China

**Keywords:** Aldose reductase inhibitor, antioxidant activity, 2-phenoxypyrido[3,2-*b*]pyrazin-3(4*H*)-one

## Abstract

To develop multifunctional aldose reductase (AKR1B1) inhibitors for anti-diabetic complications, a novel series of 2-phenoxypyrido[3,2-*b*]pyrazin-3(4*H*)-one derivatives were designed and synthesised. Most of the derivatives were found to be potent and selective against AKR1B1, and 2-(7-chloro-2-(3,5-dihydroxyphenoxy)-3-oxopyrido[3,2-*b*]pyrazin-4(3*H*)-yl) acetic acid (**4k**) was the most active with an IC_50_ value of 0.023 µM. Moreover, it was encouraging to find that some derivatives showed strong antioxidant activity, and among them, the phenolic 3,5-dihydroxyl compound **4l** with 7-bromo in the core structure was proved to be the most potent, even comparable to that of the well-known antioxidant Trolox. Thus the results suggested success in the construction of potent and selective AKR1B1 inhibitors with antioxidant activity.

## Introduction

**Figure 1.**
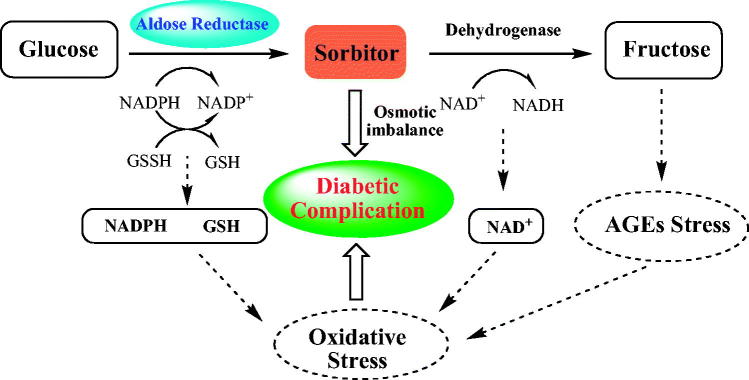
Polyol pathway and pathogenesis of diabetic complications.

Diabetes mellitus (DM) is a metabolic disorder resulting from defects in insulin secretion, insulin action, or both^1^. People suffering from DM are vulnerable to chronic diabetic complications, including neuropathy, retinopathy, nephropathy, and cataracts, which are the major menace to diabetic patients^2^^,^^3^. Increasing clinical research indicated that the abnormal polyol pathway flux of blood glucose is obviously related to pathogenesis of diabetes complications^4^. Normally, the blood glucose is predominantly converted to glucose-6-phosphate by hexokinase and then enters the glycolytic pathway. However, at high glucose concentration, specifically in diabetics, the combining capacity of aldose reductase (AKR1B1) to glucose is motivated and about one-third of the total glucose is metabolised through the polyol pathway in tissues such as lens, kidney, retina, and peripheral nerves^5–7^. In polyol pathway (Figure 1), AKR1B1 is the first enzyme and catalyses the reduction of glucose by NADPH to sorbitol, which can, in turn, be oxidised by the enzyme sorbitol dehydrogenase with concomitant reduction of NAD^+^. In the tissues implicated in these pathologies, the increased polyol pathway flux would directly cause the accumulation of sorbitol, which is hard to penetrate through cellular membranes, resulting in osmotic imbalance, cell swelling, and membrane permeability changes. All these AKR1B1-mediated biochemical alterations contribute to the pathogenesis of diabetic complications^8–10^. Therefore, design and synthesis aldose reductase inhibitors (ARIs) to inhibit the activity of AKR1B1 and further regulate the polyol pathway of glucose are likely to be a promising therapy to prevent the development of diabetic complications.

**Figure 2.**
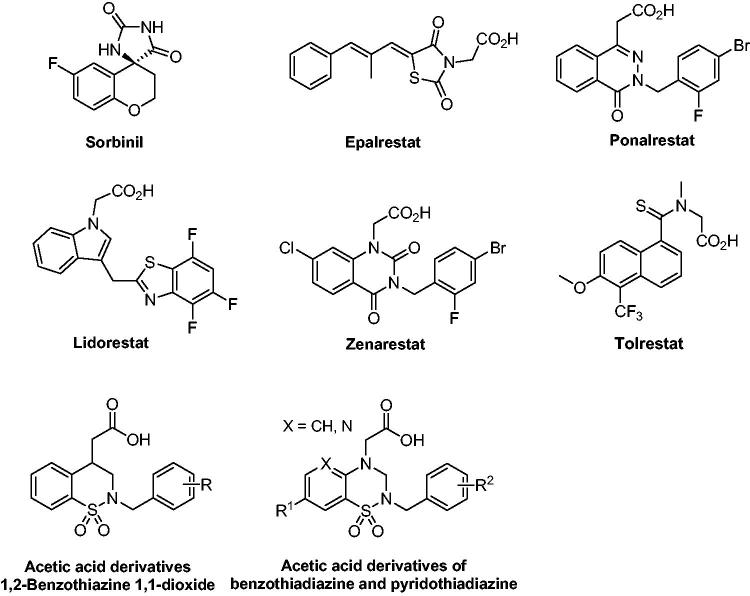
Structures of ARIs.

Numerous variously ARIs (Figure 2) have been developed and some of them are endowed with excellent inhibitory activity^11–15^. However, most of the ARIs have failed clinically due to inadequate efficacies or pharmacokinetic drawbacks. Although the underlying mechanism of the low efficacy is unclear at present, it is speculated that only inhibiting the accumulation of sorbitol is not enough to prevent and treat pathological changes in all the tissues. As the reduction of glucose consumes NADPH, which is required for regenerating reduced glutathione (GSH), this could induce intracellular oxidative stress (Figure 1). In addition, the abnormal decrease of NAD^+^ gives rise to changes in cellular redox potentials and the activity of enzymes such as nitric oxide synthase (NOS) and GSH reductase would further exacerbate intracellular oxidative stress^16–18^. The pathogenesis of diabetic complications and hyperglycaemia-induced oxidative stress always promote to each other. Thus, it will be particularly important to develop ARIs having antioxidant activity, which keeps the enzyme in its reduced form and decreases the damage due to oxidative stress, and this could enhance the overall efficacy of ARIs targeted at diabetic complications.

Based on our previous work of structurally different ARIs^19–22^, we have engaged in preparing a novel series of 2-phenoxypyrido[3,2-*b*]pyrazin-3(4*H*)-one derivatives as the new multifunctional ARIs with combined AKR1B1 inhibition and antioxidant activity. This study will focus on the further optimisation of the C2 side chain and the introduction of halogen substituent to the C7 position of the core structure.

## Chemistry

**Scheme 1.**
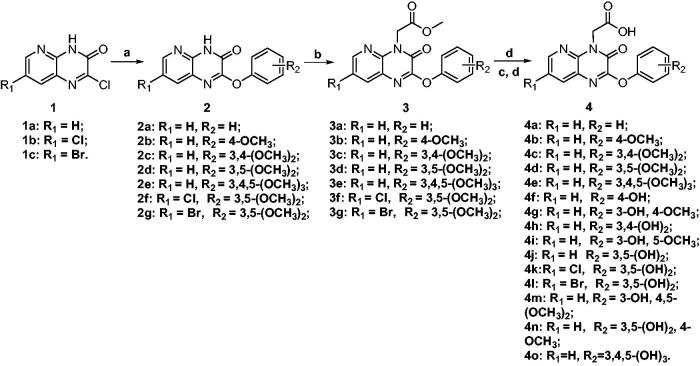
a: PhOH, DMF, K_2_CO_3_, 75 °C; b: BrCH_2_COOCH_3_, K_2_CO_3_, CH_3_CN, 55 °C; c: AlCl_3_, CH_2_Cl_2_, 45 °C; d: LiOH, H_2_O, THF, r.t., then 0.1 N HCl.

The designed compounds with an acetic acid substituent at N4 position and a variety of phenoxyl substituents at C2 position of the pyrido[2,3-*b*]pyrazin-3(4*H*)-one scaffold were obtained by the syntheses starting from compounds **1a–c**, which was prepared according to our previous reported methodology^20^^,^^21^. As shown in Scheme 1, compounds **1a–c** were firstly reacted with different phenolate to obtain compounds **2a–g**. Then the phenol methyl ethers **2a–g** were alkylated with methyl bromoacetate at the N4 position to form **3a–g** as key intermediates. Direct hydrolysis of **3a–e** with lithium hydroxide yielded compounds **4a–e**, and demethylation of partial or all the methoxyl with AlCl_3_ followed by hydrolysis of **3b–g** gave the target compounds **4f–o**. The detailed experimental data was depicted in the supplemental material.

## Results and discussion

All new synthetic compounds **4a–o** were tested for their potential inhibitory activity of AKR1B1 isolated from rat lenses. Besides, the inhibitory activity of aldehyde reductase (AKR1A1) isolated from rat kidneys was also investigated to evaluate the selectivity for AKR1B1. The enzyme AKR1A1 is closely related to AKR1B1, and plays an important role in physiological detoxification. The validity of the inhibition results was assessed with respect to epalrestat as a positive ARI. Additionally, the antioxidant activity was assessed by using the model reaction with the stable free radical of 2,2-diphenyl-1-picrylhydrazyl (DPPH) according to the modified method, which was first employed by Blois^23^.

### Inhibition of enzymes

As shown in Table 1, most of the 2-phenoxypyrido[3,2-*b*]pyrazin-3(4*H*)-one derivatives showed significant AKR1B1 inhibition and selectivity. Of all the compounds, 2–(7-chloro-2–(3,5-dihydroxyphenoxy)-3-oxopyrido[3,2-*b*]pyrazin-4(3*H*)-yl)acetic acid (**4k**) was the most active having an IC_50_ value of 0.023 µM and was more potent than epalrestat. In contrast, compound **4a**, which has no structural modification on the core structure and C2 phenoxyl side chain, was the lowest effective with an IC_50_ value of 8.596 µM. It is encouraging to find that introduction of phenolic hydroxyl or methoxyl to the C2 phenoxyl ring of **4a** could enhance the inhibitory activity. Compounds **4b–e**, **4g**, **4i**, **4m,** and **4n** containing one or more methoxyl on the C2-phenoxyl side chain displayed relatively low AKR1B1 inhibition with IC_50_ values ranging from 2.357 to 7.569 µM. Further demethylation of all methoxyl group leading to compounds **4f**, **4h**, **4j,** and **4o** showed an obvious enhancement in AKR1B1 inhibition with IC_50_ values in the range between 0.087 and 0.859 µM. The phenolic hydroxyl in C2 phenoxyl ring had an effect on AKR1B1 inhibition with the rank order of 3,5-(OH)_2_>4-OH > 3,4-(OH)_2_>3,4,5-(OH)_3_>3,5-(OH)_2_, 4-OCH_3_>3-OH, 4-OCH_3_>3-OH, 5-OCH_3_>3-OH, 4,5-(OCH_3_)_2_. Furthermore, the halogen substituent (Cl or Br) was introduced at the C7 position of compound **4j** giving **4k** and **4l**, which largely increased the inhibitory activity. Analysis of the structure–activity relationship (SAR) indicated that introduction of both phenolic hydroxyl to the C2 phenoxyl ring and halogen substituent at the C7 position of the core enhanced the AKR1B1 inhibitory activity. In addition, all compounds were also tested for their inhibition ability against AKR1A1, and showed low activity with IC_50_ values more than 15 µM, demonstrating good selectivity for AKR1B1.

**Table 1.** Enzyme inhibition activity of 2–(3-oxo-2-phenoxypyrido[3,2-*b*]pyrazin-4(3*H*)- yl)acetic acid derivatives.


	Substituent		Inhibition/AKR1B1^a^	Inhibition/AKR1A1^a^
Compd.	R_1_	R_2_	IC_50_/μM	IC_50_/μM
**4a**	H	H	8.569 ± 0.523	26.914 ± 1.453
**4b**	H	4-OCH_3_	2.684 ± 0.199	19.751 ± 1.323
**4c**	H	3,4-(OCH_3_)_2_	5.678 ± 0.608	17.623 ± 1.022
**4d**	H	3,5-(OCH_3_)_2_	4.297 ± 0.288	21.514 ± 1.807
**4e**	H	3,4,5-(OCH_3_)_3_	7.569 ± 0.431	20.874 ± 1.211
**4f**	H	4-OH	0.121 ± 0.010	15.942 ± 0.781
**4g**	H	3-OH, 4-OCH_3_	2.500 ± 0.203	19.278 ± 1.812
**4h**	H	3,4-(OH)_2_	0.305 ± 0.022	16.874 ± 0.692
**4i**	H	3-OH, 5-OCH_3_	2.853 ± 0.031	15.142 ± 0.924
**4j**	H	3,5-(OH)_2_	0.087 ± 0.007	20.812 ± 1.498
**4k**	Cl	3,5-(OH)_2_	0.023 ± 0.003	18.357 ± 1.340
**4l**	Br	3,5-(OH)_2_	0.056 ± 0.005	19.812 ± 1.407
**4m**	H	3-OH, 4,5-(OCH_3_)_2_	4.531 ± 0.258	31.557 ± 3.061
**4n**	H	3,5-(OH)_2_, 4-OCH_3_	2.357 ± 0.184	25.786 ± 1.650
**4o**	H	3,4,5-(OH)_3_	0.859 ± 0.058	21.668 ± 1.473
Epalrestat	–	–	0.089 ± 0.010	55.712 ± 3.287

^a^IC_50_ (95% CL) values represent the concentration of the tested compounds required to decrease enzymatic activity by 50%.

### The antioxidant activity

**Figure 3.**
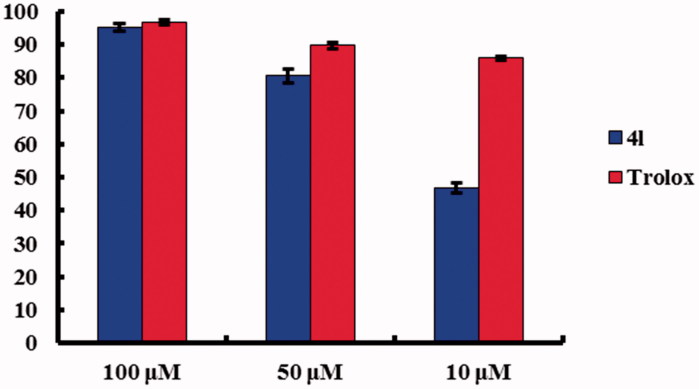
Comparison on DPPH radical scavenging activity.

The antioxidant activity of all synthesised compounds was determined with the DPPH model reaction and 6-hydroxy-2,5,7,8-chroman-2-carboxylic acid (Trolox) was employed as a positive control (Table 2). Most of the derivatives containing phenolic hydroxyl showed good DPPH radical scavenging activity ranging from 21.5 to 80.6% at the concentration of 50 µM. Of all tested compounds, **4l** with 3,5-dihydroxyl on the C2 phenoxyl ring and 7-bromo on the core structure showed the best scavenging activity, that is, 95.3, 80.6, and 46.8% at concentrations of 100, 50, and 10 µM, respectively, which had an almost similar activity compared with Trolox at high concentrations indicating the potency of antioxidant (Figure 3). SAR study of compounds **4b** *vs.* **4f**, **4c** *vs.* **4h**, **4d** *vs.* **4j**, and **4e** *vs.* **4o** indicated that the substitution of methoxyl with phenolic hydroxyl observably enhanced the radical scavenging activity. Regarding the position of phenolic hydroxyl on the C2 phenoxyl side chain, the 3,5-dihydroxyl substituent was the most effective in activity enhancement when comparing all the phenolic hydroxyl derivatives. However, comparison of compounds **4j**, **4k**, and **4l** revealed that the C7-halogen substituent had little impact on the radical scavenging activity.

**Table 2.** Antioxidant activity of 2–(3-oxo-2-phenoxypyrido[3,2-*b*]pyrazin-4(3*H*) -yl)acetic acid derivatives.


Compd.	Substituent		DPPH/sca%		
	R_1_	R_2_	100 μM	50 μM	10 μM
**4a**	H	H	6.9 ± 1.2	–	–
**4b**	H	4-OCH_3_	18.9 ± 0.8	–	–
**4c**	H	3,4-(OCH_3_)_2_	21.2 ± 1.5	–	–
**4d**	H	3,5-(OCH_3_)_2_	25.9 ± 1.3	–	–
**4e**	H	3,4,5-(OCH_3_)_3_	17.8 ± 0.9	–	–
**4f**	H	4-OH	37.7 ± 1.0	–	–
**4g**	H	3-OH, 4-OCH_3_	54.8 ± 1.6	21.5 ± 1.2	–
**4h**	H	3,4-(OH)_2_	74.6 ± 1.4	44.1 ± 2.3	–
**4i**	H	3-OH, 5-OCH_3_	78.4 ± 0.7	45.2 ± 1.8	–
**4j**	H	3,5-(OH)_2_	95.0 ± 0.9	77.2 ± 1.5	45.3 ± 1.9
**4k**	Cl	3,5-(OH)_2_	94.8 ± 1.1	76.4 ± 0.9	49.1 ± 2.4
**4l**	Br	3,5-(OH)_2_	95.3 ± 1.2	80.6 ± 2.0	46.8 ± 1.5
**4m**	H	3-OH, 4,5-(OCH_3_)_2_	56.9 ± 1.8	35.9 ± 1.1	–
**4n**	H	3,5-(OH)_2_, 4-OCH_3_	65.7 ± 0.9	40.8 ± 1.6	–
**4o**	H	3,4,5-(OH)_3_	88.5 ± 1.4	65.2 ± 1.9	23.4 ± 2.0
Trolox		–	96.7 ± 0.8	89.8 ± 1.0	85.9 ± 0.6

### Molecular docking

**Figure 4.**
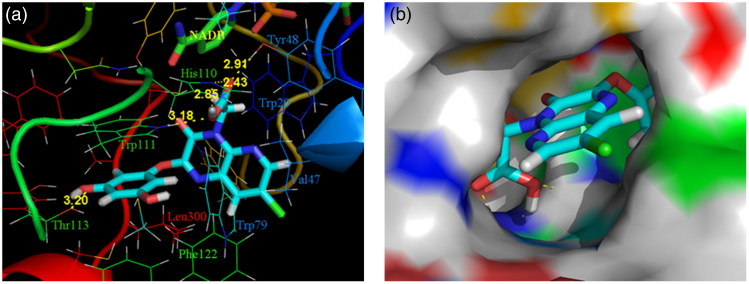
Docking of **4k** into the active site of AKR1B1. (a) The protein structure is shown in ribbon and tube representation with selected residues labeled and shown in line representation, ligand, and NADP are shown as stick models. The docked pose of **4k** is shown in cyan (C), red (O), blue (N), and green (Cl). Hydrogen bonds are shown as yellow dashed lines. (b) Protein residues are in surface representation.

Compound **4k** endowed with excellent activities both in the AKR1B1 inhibition and antioxidant reaction was docked with the conformation of the human AKR1B1/NADP^+^/lidorestat complex (PDB code: 1Z3N), to understand the mechanistic details and the above-described SARs. As shown in Figure 4, compound **4k** fitted well to the active site of AKR1B1. The carboxylate group was inserted deeply in the anion binding site by forming tight hydrogen-bonding interactions with Tyr48 (2.91 Å), Trp111 (3.18 Å), and His110 (2.85 and 2.43 Å) and engaging in a stabilizing electrostatic interaction with the positively charged nicotinamide moiety of the NADP cofactor (N–O = 4.12 Å). Besides, the 3-hydroxyl oxygen atom of the C2 phenoxyl side chain formed an additional hydrogen bond with the side chain of Thr113 (3.20 Å), confirming the importance of phenolic hydroxyl on the activity enhancement of AKR1B1 inhibition. Moreover, the 3,5-dihydroxyphenoxyl ring of the C2 side chain was well placed into the specificity pocket and paralleled to the indole ring of Trp111 forming a stable stacking interaction. Meanwhile, the pyrido[2,3-*b*]pyrazine-3(4*H*)-one core structure matched very well the hydrophobic pocket and the 7-chloro substituent pointed towards the opening of the active cleft.

## Conclusion

In conclusion, a series of novel ARI candidates (**4a–o**) based on pyrido[2,3-*b*]pyrazin-3(4*H*)-one core were synthesised and biologically evaluated for AKR1B1 inhibition and selectivity, as well as anti-oxidative properties through DPPH radical scavenging test. All compounds exhibited excellent AKR1B1 inhibitory activity in the IC_50_ range of 0.023 to 8.569 µM. Therein, compounds **4j**, **4k,** and **4l** containing phenolic 3,5-dihydroxyl on the C2 phenoxyl side chain were not only sufficient to inhibit AKR1B1 but also effective for DPPH radical scavenging, which indicated success in the development of potent ARIs with antioxidant activity. Compound **4k** was the most potent against AKR1B1 and much less active against AKR1A1 suggesting distinguished selectivity, while compound **4l** showed the best DPPH scavenging activity even comparable with Trolox at high concentrations. The SAR studies indicated that the combination of phenolic 3,5-dihydroxyl in the C2-phenoxy group and C7-halogen (Cl or Br) in the core structure largely increased the AKR1B1 inhibitory activity, and the 3,5-dihydroxyl substituent contributed greatly to the activity enhancement of anti-oxidation when comparing all the phenolic hydroxyl derivatives, which provided a beneficial strategy for the discovery of new potent ARIs with antioxidant activity.

## Supplementary Material

Supplemental Material
