# Supplementary material for: Novel 2-phenoxypyrido[3,2-b]pyrazin-3(4H)-one derivatives as potent and selective aldose reductase inhibitors with antioxidant activity
Source: J Enzyme Inhib Med Chem. 2019 Jul 26;34(1):1368–72. doi: 10.1080/14756366.2019.1643336 (PMC6711126; doi:10.1080/14756366.2019.1643336)
Supplement: Supplemental Material [file IENZ_A_1643336_SM1125.doc]

**Novel 2-phenoxypyrido[3,2-*b*]pyrazin-3(4*H*)-one derivatives as potent and selective aldose reductase inhibitors with antioxidant activity**

Xin Hao*^a,b^*, Gang Qi*^c^*, Hongxing Ma*^c^*, Changjin Zhu*^b^* and Zhongfei Han*^c,b^**

*^a^ The State Key Laboratory of Medicinal Chemical Biology, Nankai University, Tianjin, China; ^b^ Department of Applied Chemistry, Beijing Institute of Technology, Beijing, China; ^c^ Faculty of chemistry and chemical engineering, Yancheng Institute of Technology, Yancheng, China.*

*Corresponding author. *hanzhongfeibit@126.com*

**Experimental section**

**Chemistry**

All reactions were checked by TLC on silica gel Merck 60F254. The ^1^H and ^13^C NMR spectra were recorded on a Bruker Advance 400 spectrometer (400 MHz, Bruker (Beijing) Technologies and Services Co., Ltd.). Chemical shifts are given in δ/ppm units (ppm) relative to internal standard TMS and refer to CDCl_3_ or DMSO-*d*_6_ solutions. Melting points were recorded on an X-4 microscopic melting point apparatus and are uncorrected. The following HPLC methods were used to determine the purity of acetic acid derivatives using a Hitachi D-2000 Elite HPLC system. All target acetic acid derivatives tested in biological assays were > 95% pure with the following method: Inertsil ODS-2 250 mm × 10 mm, 5 mm column; mobile phase: CH_3_CN (0.1% TFA)/CH_3_OH = 75/25, for 8 min; room temperature; flow rate: 1 mL/min; detection at λ = 254 nm.

**General procedure for synthesis of 2a-g**

A mixture of K_2_CO_3_ (1 mmol), DMF (10 mL) and phenols (1.1 mmol) was added in 100 mL flask, then pyrido[2,3-b] pyrazin-3(4H)-one was added. The reaction mixture was stirred at 75^o^C for 12 h and cooled to room temperature. Then H_2_O (70 mL) and 5ml 1N HCl was added. The residue was filtered off, washed with H_2_O, and recrystallized to give compounds **2a-g**.

**2-phenoxypyrido[3,2-*b*]pyrazin-3(4*H*)-one (2a)**

Yellow solid (0.79 g, 69.6%); purity: 97.71%; mp: 211-223^o^C; ^1^H NMR (400 MHz, [D_6_]DMSO): δ/ppm = 8.11 (m, 1H), 8.06 (s, 1H), 8.01-7.98 (m, 2H), 7.66-7.40 (m, 2H), 7.29 (m, 1H), 7.01 (m, 1H), 6.62 (m, 1H); ^13^C NMR (100 MHz, [D_6_]DMSO) δ/ppm = 159.5, 156.9, 148.2, 146.7, 133.5, 131.2, 130.2, 130.1, 124.5, 116.5, 113.5, 108.2.

**2-(4-methoxyphenoxy)pyrido[3,2-*b*]pyrazin-3(4*H*)-one (2b)**

Yellow solid (0.79 g, 71.4%); purity: 98.56%; mp: 219-232^o^C; ^1^H NMR (400 MHz, [D_6_]DMSO): δ/ppm = 8.11 (m, 1H), 8.06 (s, 1H), 8.03-7.98 (m, 2H), 7.61-7.40 (m, 2H), 7.29 (m, 1H), 7.01 (m, 1H), 6.62 (m, 1H), 3.82 (s, 3H); ^13^C NMR (100 MHz, [D_6_]DMSO) δ/ppm = 159.5, 156.9, 148.2, 146.7, 133.5, 131.2, 130.2, 130.1, 124.5, 116.5, 113.5, 108.2, 55.8.

**2-(3,4-dimethoxyphenoxy)pyrido[3,2-*b*]pyrazin-3(4*H*)-one (2c)**

Yellow solid (0.89 g, 81.2%); purity: 98.21%; mp: 231-239^o^C; ^1^H NMR (400 MHz, [D_6_]DMSO): δ/ppm = 8.11 (m, 1H), 8.06 (s, 1H), 8.03-7.98 (m, 2H), 7.61-7.40 (m, 2H), 7.29 (m, 1H), 7.01 (m, 1H), 6.62 (m, 1H), 3.82 (s, 3H), 3.75 (s, 3H); ^13^C NMR (100 MHz, [D_6_]DMSO) δ/ppm = 159.5, 156.9, 148.2, 146.7, 133.5, 131.2, 130.2, 130.1, 124.5, 116.5, 113.5, 108.2, 55.8, 54.9.

**2-(3,5-dimethoxyphenoxy)pyrido[3,2-*b*]pyrazin-3(4*H*)-one (2d)**

Yellow solid (0.75 g, 64.9%); purity: 96.59%; mp: 231-241^o^C; ^1^H NMR (400 MHz, [D_6_]DMSO): δ/ppm = 8.11 (m, 1H), 8.06 (s, 1H), 8.03-7.98 (m, 2H), 7.61-7.40 (m, 2H), 7.29 (m, 1H), 7.01 (m, 1H), 6.62 (m, 1H), 3.79 (s, 6H); ^13^C NMR (100 MHz, [D_6_]DMSO) δ/ppm = 159.5, 156.9, 148.2, 146.7, 133.5, 131.2, 130.2, 130.1, 124.5, 116.5, 113.5, 108.2, 55.8, 55.2.

**2-(3,4,5-trimethoxyphenoxy)pyrido[3,2-*b*]pyrazin-3(4*H*)-one (2e)**

Yellow solid (0.84 g, 83.2%); purity: 98.27%; mp: 227-234^o^C; ^1^H NMR (400 MHz, [D_6_]DMSO): δ/ppm = 8.11 (m, 1H), 8.06 (s, 1H), 8.03-7.98 (m, 2H), 7.61-7.40 (m, 2H), 7.29 (m, 1H), 7.01 (m, 1H), 6.62 (m, 1H), 3.79 (s, 6H) 3.72 (s, 3H) ; ^13^C NMR (100 MHz, [D_6_]DMSO) δ/ppm = 159.5, 156.9, 148.2, 146.7, 133.5, 131.2, 130.2, 130.1, 124.5, 116.5, 113.5, 108.2, 55.8, 55.2, 54.9.

**7-chloro-2-(3,5-dimethoxyphenoxy)pyrido[3,2-*b*]pyrazin-3(4*H*)-one (2f)**

Yellow solid (0.64 g, 89.7%); purity: 98.47%; mp: 221-234^o^C; ^1^H NMR (400 MHz, [D_6_]DMSO): δ/ppm = 8.11 (m, 1H), 8.06 (s, 1H), 8.03-7.98 (m, 2H), 7.61-7.40 (m, 2H), 7.29 (m, 1H), 7.01 (m, 1H), 6.62 (m, 1H), 3.79 (s, 6H); ^13^C NMR (100 MHz, [D_6_]DMSO) δ/ppm = 159.5, 156.9, 148.2, 146.7, 133.5, 131.2, 130.2, 130.1, 124.5, 116.5, 113.5, 108.2, 55.8, 55.2.

**7-bromo-2-(3,5-dimethoxyphenoxy)pyrido[3,2-*b*]pyrazin-3(4*H*)-one (2g)**

Yellow solid (0.99 g, 91.4%); purity: 97.54%; mp: 229-238^o^C; ^1^H NMR (400 MHz, [D_6_]DMSO): δ/ppm = 8.11 (m, 1H), 8.06 (s, 1H), 8.03-7.98 (m, 2H), 7.61-7.40 (m, 2H), 7.29 (m, 1H), 7.01 (m, 1H), 6.62 (m, 1H), 3.79 (s, 6H); ^13^C NMR (100 MHz, [D_6_]DMSO) δ/ppm = 159.5, 156.9, 148.2, 146.7, 133.5, 131.2, 130.2, 130.1, 124.5, 116.5, 113.5, 108.2, 55.8, 55.2.

**General procedure for synthesis of 3a-g**

A mixture of the compounds **2a-g**, K_2_CO_3_ (1 mmol), and methyl bromoacetate (1.1 mmol) in CH_3_CN (30 mL) was stirred at 55^o^C for 2 h and then filtered. After evaporation of the solvent in vacuo, the residue was purified by column chromatography to furnish compound **3a-g**.

**Methyl 2-(3-oxo-2-phenoxypyrido[3,2-*b*]pyrazin-4(3*H*)-yl)acetate (3a)**

Yellow solid (0.35 g, 71.2%); purity: 95.91%; mp: 241-250^o^C; ^1^H NMR (400 MHz, [D_6_]DMSO): δ/ppm = 8.11 (m, 1H), 8.06 (s, 1H), 8.01-7.98 (m, 2H), 7.66-7.40 (m, 2H), 7.29 (m, 1H), 7.01 (m, 1H), 6.62 (m, 1H) 3.92 (s, 2H), 3.68 (s, 3H); ^13^C NMR (100 MHz, [D_6_]DMSO) δ/ppm = 169.5, 159.5, 156.9, 148.2, 146.7, 133.5, 131.2, 130.2, 130.1, 124.5, 116.5, 113.5, 108.2, 51.6, 51.3.

**Methyl 2-(2-(4-methoxyphenoxy)-3-oxopyrido[3,2-*b*]pyrazin-4(3*H*)-yl)acetate (3b)**

Yellow solid (0.32 g, 68.4%); purity: 96.42%; mp: 246-252^o^C; ^1^H NMR (400 MHz, [D_6_]DMSO): δ/ppm = 8.11 (m, 1H), 8.06 (s, 1H), 8.03-7.98 (m, 2H), 7.61-7.40 (m, 2H), 7.29 (m, 1H), 7.01 (m, 1H), 6.62 (m, 1H), 3.92 (s, 2H), 3.82 (s, 3H), 3.68 (s, 3H); ^13^C NMR (100 MHz, [D_6_]DMSO) δ/ppm =169.4 159.5, 156.9, 148.2, 146.7, 133.5, 131.2, 130.2, 130.1, 124.5, 116.5, 113.5, 108.2, 55.8, 51.6, 51.3.

**Methyl 2-(2-(3,4-dimethoxyphenoxy)-3-oxopyrido[3,2-*b*]pyrazin-4(3*H*)-yl)acetate (3c)**

Yellow solid (0.47 g, 64.8%); purity: 97.48%; mp: 238-241^o^C; ^1^H NMR (400 MHz, [D_6_]DMSO): δ/ppm = 8.11 (m, 1H), 8.06 (s, 1H), 8.03-7.98 (m, 2H), 7.61-7.40 (m, 2H), 7.29 (m, 1H), 7.01 (m, 1H), 6.62 (m, 1H), 3.92 (s, 2H), 3.82 (s, 3H), 3.75 (s, 3H) 3.68 (s, 3H); ^13^C NMR (100 MHz, [D_6_]DMSO) δ/ppm = 159.5, 156.9, 148.2, 146.7, 133.5, 131.2, 130.2, 130.1, 124.5, 116.5, 113.5, 108.2, 55.8, 54.9, 51.6, 51.3.

**Methyl 2-(2-(3,5-dimethoxyphenoxy)-3-oxopyrido[3,2-*b*]pyrazin-4(3*H*)-yl)acetate (3d)**

Yellow solid (0.29 g, 75.9%); purity: 98.81%; mp: 235-242^o^C; ^1^H NMR (400 MHz, [D_6_]DMSO): δ/ppm = 8.11 (m, 1H), 8.06 (s, 1H), 8.03-7.98 (m, 2H), 7.61-7.40 (m, 2H), 7.29 (m, 1H), 7.01 (m, 1H), 6.62 (m, 1H), 3.92 (s, 2H), 3.79 (s, 6H), 3.68 (s, 3H); ^13^C NMR (100 MHz, [D_6_]DMSO) δ/ppm = 159.5, 156.9, 148.2, 146.7, 133.5, 131.2, 130.2, 130.1, 124.5, 116.5, 113.5, 108.2, 55.8, 55.2, 51.6, 51.3.

**Methyl2-(3-oxo-2-(3,4,5-trimethoxyphenoxy)pyrido[3,2-*b*]pyrazin-4(3*H*)-yl) acetate (3e)**

Yellow solid (0.42 g, 55.7%); purity: 99.18%; mp: 236-245^o^C; ^1^H NMR (400 MHz, [D_6_]DMSO): δ/ppm = 8.11 (m, 1H), 8.06 (s, 1H), 8.03-7.98 (m, 2H), 7.61-7.40 (m, 2H), 7.29 (m, 1H), 7.01 (m, 1H), 6.62 (m, 1H), 3.92 (s, 2H), 3.79 (s, 6H), 3.72 (s, 3H), 3.68 (s, 3H); ^13^C NMR (100 MHz, [D_6_]DMSO) δ/ppm = 159.5, 156.9, 148.2, 146.7, 133.5, 131.2, 130.2, 130.1, 124.5, 116.5, 113.5, 108.2, 55.8, 55.2, 54.9, 51.6, 51.3.

**Methyl-2-(7-chloro-2-(3,5-dimethoxyphenoxy)-3-oxopyrido[3,2-*b*]pyrazin-4(3*H*)-yl)acetate (3f)**

Yellow solid (0.34 g, 84.7%); purity: 96.91%; mp: 249-257^o^C; ^1^H NMR (400 MHz, [D_6_]DMSO): δ/ppm = 8.11 (m, 1H), 8.06 (s, 1H), 8.03-7.98 (m, 2H), 7.61-7.40 (m, 2H), 7.29 (m, 1H), 7.01 (m, 1H), 6.62 (m, 1H), 3.92 (s, 2H), 3.79 (s, 6H), 3.68 (s, 3H); ^13^C NMR (100 MHz, [D_6_]DMSO) δ/ppm = 159.5, 156.9, 148.2, 146.7, 133.5, 131.2, 130.2, 130.1, 124.5, 116.5, 113.5, 108.2, 55.8, 55.2, 51.6, 51.3.

**Methyl-2-(7-bromo-2-(3,5-dimethoxyphenoxy)-3-oxopyrido[3,2-*b*]pyrazin-4(3*H*)-yl)acetate (3g)**

Yellow solid (0.49 g, 82.4%); purity: 97.48%; mp: 255-262^o^C; ^1^H NMR (400 MHz, [D_6_]DMSO): δ/ppm = 8.11 (m, 1H), 8.06 (s, 1H), 8.03-7.98 (m, 2H), 7.61-7.40 (m, 2H), 7.29 (m, 1H), 7.01 (m, 1H), 6.62 (m, 1H), 3.92 (s, 2H), 3.79 (s, 6H), 3.68 (s, 3H); ^13^C NMR (100 MHz, [D_6_]DMSO) δ/ppm = 159.5, 156.9, 148.2, 146.7, 133.5, 131.2, 130.2, 130.1, 124.5, 116.5, 113.5, 108.2, 55.8, 55.2, 51.6, 51.3.

**General procedure for synthesis (4a-o)**

To a suspension of anhydrous AlCl_3_ (10 mmol) in anhydrous DCM (25 mL) was added the solution of **3b-g** (1 mmol) in anhydrous DCM (10 mL) at 0°C. The reaction vessel was heated to 45°C for 24 hours, cooled to room temperature and poured into ice-cold water (15 mL). The aqueous phase was extracted with ethyl acetate (3 x 25 mL), and the combined organics were washed with brine, dried, filtered, and concentrated. The residue was purified by column chromatography, and then the obtained residue (1 mmol) in THF (2 mL) was added lithium hydroxide (3 mmol) in H_2_O (2 mL), stirred at room temperature for 2 h. The reaction mixture was evaporated to dryness and dissolved in H_2_O. Then the mixture was washed with EtOAc (30 mL), and the aqueous layer was acidified to pH 2-3 with appropriate 1 M HCl. The precipitate was washed with water to give compounds **4a-o** respectively.

**2-(3-oxo-2-phenoxypyrido[3,2-*b*]pyrazin-4(3*H*)-yl)acetic acid (4a)**

Yellow solid (0.16 g, 47.5%); purity: 98.21%; mp: 239-244^o^C; ^1^H NMR (400 MHz, [D_6_]DMSO): δ/ppm = 11.0 (s, H), 8.11 (m, 1H), 8.06 (s, 1H), 8.01-7.98 (m, 2H), 7.66-7.40 (m, 2H), 7.29 (m, 1H), 7.01 (m, 1H), 6.62 (m, 1H) 3.92 (s, 2H); ^13^C NMR (100 MHz, [D_6_]DMSO) δ/ppm = 169.5, 159.5, 156.9, 148.2, 146.7, 133.5, 131.2, 130.2, 130.1, 124.5, 116.5, 113.5, 108.2, 51.6.

**2-(2-(4-methoxyphenoxy)-3-oxopyrido[3,2-*b*]pyrazin-4(3*H*)-yl)acetic acid (4b)**

Yellow solid (0.11 g, 61.5%); purity: 97.11%; mp: 234-242^o^C; ^1^H NMR (400 MHz, [D_6_]DMSO): δ/ppm = 11.1 (s, 1H), 8.11 (m, 1H), 8.06 (s, 1H), 8.03-7.98 (m, 2H), 7.61-7.40 (m, 2H), 7.29 (m, 1H), 7.01 (m, 1H), 6.62 (m, 1H), 3.92 (s, 2H), 3.82 (s, 3H); ^13^C NMR (100 MHz, [D_6_]DMSO) δ/ppm =169.4 159.5, 156.9, 148.2, 146.7, 133.5, 131.2, 130.2, 130.1, 124.5, 116.5, 113.5, 108.2, 55.8, 51.6.

**2-(2-(3,4-dimethoxyphenoxy)-3-oxopyrido[3,2-*b*]pyrazin-4(3*H*)-yl)acetic acid (4c)**

Yellow solid (0.12 g, 51.2%); purity: 98.44%; mp: 241-251^o^C; ^1^H NMR (400 MHz, [D_6_]DMSO): δ/ppm = 11.1 (s, 1H), 8.11 (m, 1H), 8.06 (s, 1H), 8.03-7.98 (m, 2H), 7.61-7.40 (m, 2H), 7.29 (m, 1H), 7.01 (m, 1H), 6.62 (m, 1H), 3.92 (s, 2H), 3.82 (s, 3H), 3.75 (s, 3H); ^13^C NMR (100 MHz, [D_6_]DMSO) δ/ppm = 159.5, 156.9, 148.2, 146.7, 133.5, 131.2, 130.2, 130.1, 124.5, 116.5, 113.5, 108.2, 55.8, 54.9, 51.6.

**2-(2-(3,5-dimethoxyphenoxy)-3-oxopyrido[3,2-*b*]pyrazin-4(3*H*)-yl)acetic acid (4d)**

Yellow solid (0.13 g, 59.4%); purity: 96.21%; mp: 241-247^o^C; ^1^H NMR (400 MHz, [D_6_]DMSO): δ/ppm = 11.1 (s, 1H), 8.11 (m, 1H), 8.06 (s, 1H), 8.03-7.98 (m, 2H), 7.61-7.40 (m, 2H), 7.29 (m, 1H), 7.01 (m, 1H), 6.62 (m, 1H), 3.92 (s, 2H), 3.79 (s, 6H); ^13^C NMR (100 MHz, [D_6_]DMSO) δ/ppm = 159.5, 156.9, 148.2, 146.7, 133.5, 131.2, 130.2, 130.1, 124.5, 116.5, 113.5, 108.2, 55.8, 55.2, 51.6.

**2-(3-oxo-2-(3,4,5-trimethoxyphenoxy)pyrido[3,2-*b*]pyrazin-4(3*H*)-yl)acetic acid (4e)**

Yellow solid (0.14 g, 69.4%); purity: 97.94%; mp: 235-243^o^C; ^1^H NMR (400 MHz, [D_6_]DMSO): δ/ppm = 11.1 (s, 1H); 8.11 (m, 1H), 8.06 (s, 1H), 8.03-7.98 (m, 2H), 7.61-7.40 (m, 2H), 7.29 (m, 1H), 7.01 (m, 1H), 6.62 (m, 1H), 3.92 (s, 2H), 3.79 (s, 6H), 3.72 (s, 3H); ^13^C NMR (100 MHz, [D_6_]DMSO) δ/ppm = 159.5, 156.9, 148.2, 146.7, 133.5, 131.2, 130.2, 130.1, 124.5, 116.5, 113.5, 108.2, 55.8, 55.2, 54.9, 51.6.

**2-(2-(4-hydroxyphenoxy)-3-oxopyrido[3,2-*b*]pyrazin-4(3*H*)-yl)acetic acid (4f)**

Yellow solid (0.11 g, 68.5%); purity: 96.35%; mp: 231-239^o^C; ^1^H NMR (400 MHz, [D_6_]DMSO): δ/ppm = 11.1 (s, 1H), 8.11 (m, 1H), 8.06 (s, 1H), 8.03-7.98 (m, 2H), 7.61-7.40 (m, 2H), 7.29 (m, 1H), 7.01 (m, 1H), 6.62 (m, 1H), 5.35 (s, 1H), 3.92 (s, 2H); ^13^C NMR (100 MHz, [D_6_]DMSO) δ/ppm =169.4 159.5, 156.9, 148.2, 146.7, 133.5, 131.2, 130.2, 130.1, 124.5, 116.5, 113.5, 108.2, 55.8.

**2-(2-(3-hydroxy-4-methoxyphenoxy)-3-oxopyrido[3,2-*b*]pyrazin-4(3*H*)-yl)acetic acid (4g)**

Yellow solid (0.17 g, 72.5%); purity: 97.49%; mp: 232-236^o^C; ^1^H NMR (400 MHz, [D_6_]DMSO): δ/ppm = 11.1 (s, 1H), 8.11 (m, 1H), 8.06 (s, 1H), 8.03-7.98 (m, 2H), 7.61-7.40 (m, 2H), 7.29 (m, 1H), 7.01 (m, 1H), 6.62 (m, 1H), 5.34 (s, 1H), 3.92 (s, 2H), 3.82 (s, 3H); ^13^C NMR (100 MHz, [D_6_]DMSO) δ/ppm = 159.5, 156.9, 148.2, 146.7, 133.5, 131.2, 130.2, 130.1, 124.5, 116.5, 113.5, 108.2, 55.8, 54.9.

**2-(2-(3,4-dihydroxyphenoxy)-3-oxopyrido[3,2-*b*]pyrazin-4(3*H*)-yl)acetic acid (4h)**

Yellow solid (0.14 g, 65.7%); purity: 98.91%; mp: 231-236^o^C; ^1^H NMR (400 MHz, [D_6_]DMSO): δ/ppm = 11.1 (s, 1H), 8.11 (m, 1H), 8.06 (s, 1H), 8.03-7.98 (m, 2H), 7.61-7.40 (m, 2H), 7.29 (m, 1H), 7.01 (m, 1H), 6.62 (m, 1H), 5.34 (s, 1H), 5.21 (s, 1H), 3.92 (s, 2H); ^13^C NMR (100 MHz, [D_6_]DMSO) δ/ppm = 159.5, 156.9, 148.2, 146.7, 133.5, 131.2, 130.2, 130.1, 124.5, 116.5, 113.5, 108.2, 55.8.

**2-(2-(3-hydroxy-5-methoxyphenoxy)-3-oxopyrido[3,2-*b*]pyrazin-4(3*H*)-yl)acetic acid (4i)**

Yellow solid (0.16 g, 58.1%); purity: 98.21%; mp: 229-232^o^C; ^1^H NMR (400 MHz, [D_6_]DMSO): δ/ppm = 11.1 (s, 1H), 8.11 (m, 1H), 8.06 (s, 1H), 8.03-7.98 (m, 2H), 7.61-7.40 (m, 2H), 7.29 (m, 1H), 7.01 (m, 1H), 6.62 (m, 1H), 5.35 (s, 1H), 3.92 (s, 2H), 3.79 (s, 3H); ^13^C NMR (100 MHz, [D_6_]DMSO) δ/ppm = 159.5, 156.9, 148.2, 146.7, 133.5, 131.2, 130.2, 130.1, 124.5, 116.5, 113.5, 108.2, 55.8, 55.2.

**2-(2-(3,5-dihydroxyphenoxy)-3-oxopyrido[3,2-*b*]pyrazin-4(3*H*)-yl)acetic acid (4j)**

Yellow solid (0.09 g, 54.7%); purity: 98.12%; mp: 231-237^o^C; ^1^H NMR (400 MHz, [D_6_]DMSO): δ/ppm = 11.1 (s, 1H), 8.11 (m, 1H), 8.06 (s, 1H), 8.03-7.98 (m, 2H), 7.61-7.40 (m, 2H), 7.29 (m, 1H), 7.01 (m, 1H), 6.62 (m, 1H), 5.35 (s, 2H), 3.92 (s, 2H); ^13^C NMR (100 MHz, [D_6_]DMSO) δ/ppm = 159.5, 156.9, 148.2, 146.7, 133.5, 131.2, 130.2, 130.1, 124.5, 116.5, 113.5, 108.2, 55.8.

**2-(7-chloro-2-(3,5-dihydroxyphenoxy)-3-oxopyrido[3,2-*b*]pyrazin-4(3*H*)-yl)acetic acid (4k)**

Yellow solid (0.18 g, 65.1%); purity: 98.21%; mp: 232-236^o^C; ^1^H NMR (400 MHz, [D_6_]DMSO): δ/ppm = 11.1 (s, 1H), 8.11 (m, 1H), 8.03-7.98 (m, 2H), 7.61-7.40 (m, 2H), 7.29 (m, 1H), 7.01 (m, 1H), 6.62 (m, 1H), 5.35 (s, 2H), 3.92 (s, 2H); ^13^C NMR (100 MHz, [D_6_]DMSO) δ/ppm = 159.5, 156.9, 148.2, 146.7, 133.5, 131.2, 130.2, 130.1, 124.5, 116.5, 113.5, 108.2, 55.8.

**2-(7-bromo-2-(3,5-dihydroxyphenoxy)-3-oxopyrido[3,2-*b*]pyrazin-4(3*H*)-yl)acetic acid (4l)**

Yellow solid (0.13 g, 54.0%); purity: 96.01%; mp: 233-235^o^C; ^1^H NMR (400 MHz, [D_6_]DMSO): δ/ppm = 11.1 (s, 1H), 8.11 (m, 1H), 8.03-7.98 (m, 2H), 7.61-7.40 (m, 2H), 7.29 (m, 1H), 7.01 (m, 1H), 6.62 (m, 1H), 5.35 (s, 2H), 3.92 (s, 2H); ^13^C NMR (100 MHz, [D_6_]DMSO) δ/ppm = 159.5, 156.9, 148.2, 146.7, 133.5, 131.2, 130.2, 130.1, 124.5, 116.5, 113.5, 108.2, 55.8.

**2-(2-(3-hydroxy-4,5-dimethoxyphenoxy)-3-oxopyrido[3,2-*b*]pyrazin-4(3*H*)-yl)acetic acid (4m)**

Yellow solid (0.15 g, 61.8%); purity: 97.41%; mp: 237-244^o^C; ^1^H NMR (400 MHz, [D_6_]DMSO): δ/ppm = 11.1 (s, 1H); 8.11 (m, 1H), 8.06 (s, 1H), 8.03-7.98 (m, 2H), 7.61-7.40 (m, 2H), 7.29 (m, 1H), 7.01 (m, 1H), 6.62 (m, 1H), 5.35 (s, 1H), 3.92 (s, 2H), 3.79 (s, 3H), 3.72 (s, 3H); ^13^C NMR (100 MHz, [D_6_]DMSO) δ/ppm = 159.5, 156.9, 148.2, 146.7, 133.5, 131.2, 130.2, 130.1, 124.5, 116.5, 113.5, 108.2, 55.8, 55.2, 54.9.

**2-(2-(3,5-dihydroxy-4-methoxyphenoxy)-3-oxopyrido[3,2-*b*]pyrazin-4(3*H*)-yl)acetic acid (4n)**

Yellow solid (0.14 g, 66.4%); purity: 97.11%; mp: 235-241^o^C; ^1^H NMR (400 MHz, [D_6_]DMSO): δ/ppm = 11.1 (s, 1H); 8.11 (m, 1H), 8.06 (s, 1H), 8.03-7.98 (m, 2H), 7.61-7.40 (m, 2H), 7.29 (m, 1H), 7.01 (m, 1H), 6.62 (m, 1H), 5.35 (s, 2H), 3.92 (s, 2H), 3.72 (s, 3H); ^13^C NMR (100 MHz, [D_6_]DMSO) δ/ppm = 159.5, 156.9, 148.2, 146.7, 133.5, 131.2, 130.2, 130.1, 124.5, 116.5, 113.5, 108.2, 55.8, 55.2.

**2-(3-oxo-2-(3,4,5-trihydroxyphenoxy)pyrido[3,2-*b*]pyrazin-4(3*H*)-yl)acetic acid (4o)**

Yellow solid (0.11 g, 62.8%); purity: 98.41%; mp: 241-246^o^C; ^1^H NMR (400 MHz, [D_6_]DMSO): δ/ppm = 11.1 (s, 1H); 8.11 (m, 1H), 8.06 (s, 1H), 8.03-7.98 (m, 2H), 7.61-7.40 (m, 2H), 7.29 (m, 1H), 7.01 (m, 1H), 6.62 (m, 1H), 5.35 (s, 2H), 5.23 (s, 1H), 3.92 (s, 2H); ^13^C NMR (100 MHz, [D_6_]DMSO) δ/ppm = 159.5, 156.9, 148.2, 146.7, 133.5, 131.2, 130.2, 130.1, 124.5, 116.5, 113.5, 108.2, 55.8.

**Enzyme** **assays**

The AKR1B1 inhibition activity was performed in a reaction mixture containing 0.25 mL NADPH (0.10 mM), 0.25 mL sodium phosphate buffer (pH = 6.2, 0.1 M), 0.1 mL enzyme extract, 0.15 mL deionized water, and 0.25 mL _D,L_-glyceraldehyde (10 mM) as substrate in a final volume of 1 mL. Before adding to _D,L_-glyceraldehyde, the reaction mixture was incubated at 30 ^o^C for 10 min, and then the substrate was added to start the reaction, which was monitored for 4 min.

The AKR1A1 inhibition activity was performed in a reaction mixture containing 0.25 mL NADPH (0.12 mM), 0.1 mL enzyme extract, 0.25 mL sodium phosphate buffer (pH = 7.2, 0.1 M), 0.15 mL deionized water, and 0.25 mL sodium _D_-glucuronate (20 mM) as substrate in a final volume of 1 mL. Before adding to sodium _D_-glucuronate, the reaction mixture was incubated at 37^o^C for 10 min, and then the substrate was added to start the reaction, which was monitored for 4 min.

The inhibitory activity of the newly synthesized compounds against AKR1B1 and AKR1A1 was assayed by adding 5 μL of the inhibitor solution to the reaction mixture described above. All compounds were dissolved in dimethyl sulfoxide (DMSO) and diluted with deionized H_2_O. To correct for the nonenzymatic oxidation of NADPH, the rate of NADPH oxidation in the presence of all the reaction mixture components except the substrate was subtracted from each experimental rate. The inhibitory effect of the synthetic compounds was routinely estimated at 10^-4^ M (the concentration is referenced to that of the compound in the reaction mixture). The compounds found to be active were tested at additional concentrations between 10^-4^ and 10^-8^ M, the log (dose)–response curves were then constructed from the inhibitory data, and the IC_50_ values were calculated by least-square analysis of the linear portion of the log (dose) versus response curves (0.912 < r^2^ < 0.996).

**DPPH assay**

To investigate the antiradical activity of target compounds in a homogeneous system, a method based on the scavenging of the stable free radical DPPH was employed. A 100 μL of methanolic solution of various compounds with different concentrations was added to 1 mL DPPH methanolic solution (2.5 × 10^-5^ g/mL) and 1.9 ml methanol solution to give final concentrations of 0.1, 0.05, 0.01 mM for the tested compounds, respectively. After vortexing thoroughly and leaving for 30 min at room temperature, the optical density was measured at λ 517 nm using the Shimadzu UV-1800 spectrophotometer. The tested compounds and the reference (Trolox) were dissolved in methanol, and after 240 min (steady state), the percentage of DPPH radical scavenging was determined by the equation as shown below. The experiments were performed in triplicate.

$$\begin{matrix} \begin{matrix} percentage & of & scavenging\left(\%\right) \end{matrix}=\left(1-\frac{A_{sample}-A_{blank}}{A_{control}}\right)\times100\% \\ A_{control}=DPPH+methanol \\ A_{sample}=\begin{matrix} tested & compound+DPPH+methanol \end{matrix} \\ A_{blank}=\begin{matrix} tested & compound+mathanol \end{matrix} \end{matrix}$$

**Docking studies**

Docking studies were performed using AutoDockTools. The crystal structure of human aldose reductase with bound inhibitor lidorestat retrieved from the RCSB Protein Data Bank (PDB code: 1Z3N) . All solvent molecules within the protein structure were removed for the docking procedure. The binding cavity around the anion binding site (volume of 141 Å^3^) was used for docking calculations and further modified using side chain minimization. The best ligand pose was chosen according to the docking score and each docking process took 4-6 min.

## References

1. El-Kabbani, O., et al. 2005, Structure of aldehyde reductase holoenzyme in complex with the potent aldose reductase inhibitor Fidarestat:  implications for inhibitor binding and selectivity. *J.med.chem.* 48: 5536-5542.

2. Kinoshita, T., et al. 2002, The structure of human recombinant aldose reductase complexed with the potent inhibitor zenarestat. *Acta Crystallographica Section D.* 58: 622-626.
